# Functional Amyloids Are the Rule Rather Than the Exception in Cellular Biology

**DOI:** 10.3390/microorganisms8121951

**Published:** 2020-12-09

**Authors:** Anthony Balistreri, Emily Goetzler, Matthew Chapman

**Affiliations:** Department of Molecular, Cellular and Developmental Biology, University of Michigan, Ann Arbor, MI 48109, USA; abalist@umich.edu (A.B.); goetzler@umich.edu (E.G.)

**Keywords:** pathogenic amyloids, functional amyloids, curli, fap

## Abstract

Amyloids are a class of protein aggregates that have been historically characterized by their relationship with human disease. Indeed, amyloids can be the result of misfolded proteins that self-associate to form insoluble, extracellular plaques in diseased tissue. For the first 150 years of their study, the pathogen-first definition of amyloids was sufficient. However, new observations of amyloids foster an appreciation for non-pathological roles for amyloids in cellular systems. There is now evidence from all domains of life that amyloids can be non-pathogenic and functional, and that their formation can be the result of purposeful and controlled cellular processes. So-called functional amyloids fulfill an assortment of biological functions including acting as structural scaffolds, regulatory mechanisms, and storage mechanisms. The conceptual convergence of amyloids serving a functional role has been repeatedly confirmed by discoveries of additional functional amyloids. With dozens already known, and with the vigorous rate of discovery, the biology of amyloids is robustly represented by non-pathogenic amyloids.

## 1. Introduction

Amyloids are fibril protein aggregates originally identified in 1854 by Rudolph Virchow when he observed iodine-stained plaques in abnormal brain tissues [1,2,3]. Since the nineteenth century, amyloids, and the extracellular bodies they form in human tissue, have been inextricably connected to human disease and neurological dysfunction. More recent observations have led to an expansion of the definition of amyloids to include a class of non-pathogenic aggregates [1,4]. Functional amyloids are created by organisms intentionally, in order to exploit their many useful properties [5]. In the last 20 years, the list of functional amyloid proteins has blossomed (See Table 1). This has shifted views of amyloid biology from singularly pathogenic and cytotoxic structures to a protein fold that contributes positively to cellular biology. Our thesis put forth here is that functional amyloids are the principle class of amyloids found in nature.

## 2. The Established Perspective of Amyloid Proteins

Since their discovery, amyloids have been most closely associated with human disease. The amyloid field was ushered into medical science in 1854 when the German physician Rudolph Virchow observed iodine-stained “copora amylacae” in nervous tissue [39], characterizing the bodies as being starch-like (Latin for starch is *amylum*). Seven decades later, the textile dye Congo Red was identified as a useful compound for specifically staining amyloids in histological samples taken from diseased brain tissues [40]. In the same 1927 publication, the Belgian physician Paul Divry observed that Congo Red stained Alois Alzheimer’s plaques, which he associated with presenile dementia, thereby connecting these plaques to amyloids [1,40]. Divry’s publication sparked an exploration for amyloids in histological samples that continued through the twentieth century, during which, scientists found evidence of amyloids related to both localized and systematic syndromes [1,2]. The Pras extraction method introduced in 1968 allowed for a greater biochemical and structural characterization of amyloids [1,41]. Efficient fiber extraction coupled with amino acid sequencing lead researchers to determine that amyloid fibers associated with neuropathic or systemic disorders were composed of different proteins, each with their own clinical manifestations [2].

**Figure 1:**
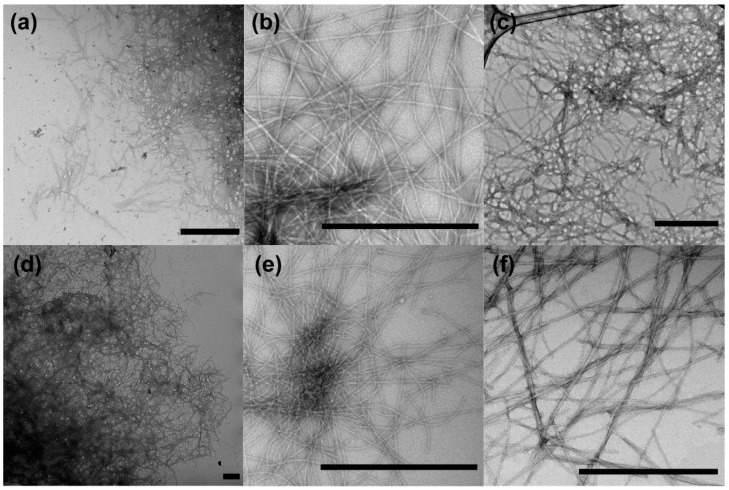
Negatively stained transmission electron micrographs of pathogenic and functional amyloid fibrils. (**a**) α-Synuclein fibers. (**b**) Aβ_1–40_ fibrils. (**c**) Amylin fibrils. (**d**) Curli fibrils. (**e**) HET-s_218–289_ fibrils. (**f**) IIKIIK fibrils associated with PSMα. Scale bars represent 200 nm. Published with permission from Robert Tycko and Neha Jain.

At the turn of the twenty-first century, several groups identified roles for amyloids other than causing disease in biology. In the late 1990s, yeast geneticists finally solved the strange issue of non-Mendelian heritable traits propagating in yeast [20,42,43,44]. The infective (i.e., prion) amyloid assemblies of the protein Sup35 was shown to have epigenetic-like control over protein expression based on the presence of the amyloid or soluble form of the protein [20,42,43,44]. In 2000, it was discovered that amyloid rodlets made up of hydrophobins allow fungi to escape an aquatic environment [21]. In the same year, amyloid proteins called chorions were identified as the major protective component surrounding silkmoth eggs [27]. In 2002, curli, the main proteinaceous component of the *E. coli* biofilm, were revealed to share biophysical characteristics with pathogenic amyloid fibers [6]. However, unlike pathogenic amyloids, these functional amyloids are not the product of stochastic protein misfolding and are often assembled via tightly regulated and controlled pathways. Since the early 2000s, many functional amyloids proteins have been discovered and described. The functional amyloids share biophysical characteristics with their pathogenic counterparts, including tinctorial properties, ability to self-assemble, and their fibrous 3D structure (Figure 1). There are now approximately 35 new functional amyloid proteins, and new ones are continuing to be described [45].

## 3. The Amyloid Fold Is Intrinsic to Polypeptides

Amyloids are highly ordered protein aggregates that are in a low energy conformation and are highly stable and resistant to denaturation [46,47]. Classically, amyloids have been defined by their biophysical characteristics, including the cross-β structure, where β-strands align perpendicular to a fibril axis [3]. Recently, the amyloid structural catalog was expanded to include the cross-α structure of phenol-soluble modulins, a functional amyloid produced by *S. aureus* [48]. Regardless, the amyloid fold consisting of repeated, structural units that engender stability and conformity is a hallmark of protein folding. It has been theorized that the amyloid fold could be a primordial structural motif, and in a prebiotic world, it represents an early form of self-propagation and information transfer [49].

**Figure 2:**
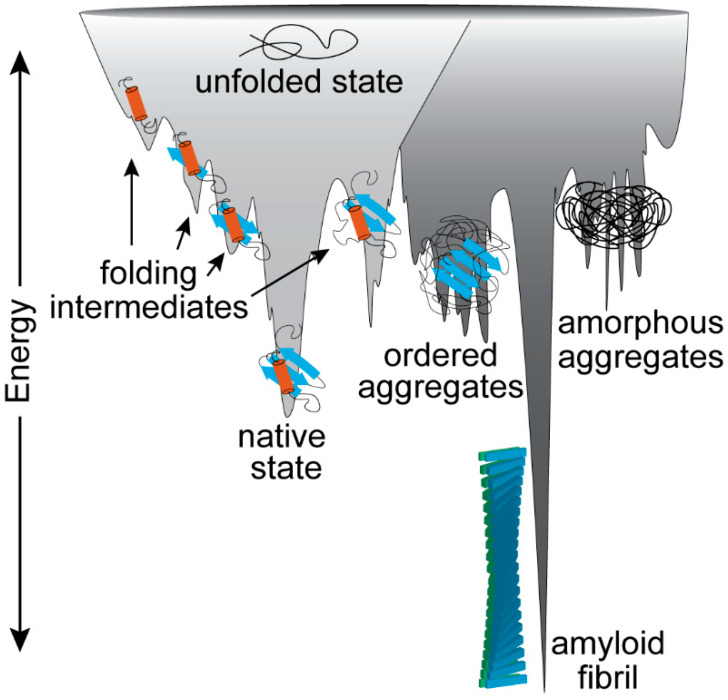
Cartoon showing the complex protein folding landscape. Nascent, unfolded proteins travel down an energy gradient seeking the lowest energy conformation. Proteins can sample various energy minima on this journey including folding intermediates and the native fold state. However, every protein has the potential to reach other energy minima, including unfolded or partially ordered aggregates. In addition, all proteins can adopt the true lowest energy state, the amyloid fibril. Adapted from Jahn and Radford [50].

It has also been suggested that the amyloid fold can be achieved by peptides, regardless of their specific amino acid composition. Anfinsen’s dogma describes protein folding, wherein the 3D structure of a fully folded protein is determined by its primary sequence [50]. Certainly, this is true of globular proteins that adopt their native fold under optimal conditions. However, the native fold is only one of several thermodynamic minima [47,51]. Indeed, even a natively folded protein exists in a metastable state and, with enough energy proteins, can fold into the amyloid-specific, cross β-sheet conformation that is the lowest energy state [52] (Figure 2). Experiments that observed human lysozyme and transthyretin adopting the amyloid fold found that partially unstable or unfolded domains were the focal point of amyloidogenesis [53,54,55]. However, even stably folded proteins, such as the SH3 domain or acylphosphatase, can form amyloid after partial denaturation [56,57,58]. Even chains of polyalanine and polyglutamate have been shown to adopt the amyloid fold during in silico modeling experiments [3,59,60]. Therefore, given the ubiquity of the amyloid conformation in protein structure, it is not surprising that amyloids are part of normal cellular biology.

## 4. Curli and Fap Are Bacterial Amyloids Whose Assembly Is Highly Orchestrated

Arguably the most-studied bacterial functional amyloids are curli and the related Fap amyloids, made by *E. coli* and *Pseudomonas* spp., respectively. Both curli and Fap amyloids are major structural components of the biofilm matrix. Biofilms are entrenched colonies of bacteria that secrete a dense matrix of polysaccharides, amyloid fibers, and nucleic acids that collectively make up the extracellular matrix [61]. Within a biofilm, bacteria can continue to grow and survive some of the harshest environments, making biofilms an important factor in bacterial infection and pathogenesis [62]. When it comes to Proteobacteria, curli amyloids are essential biofilm components, illustrated by Reichhardt et al. who estimate that curli make up as much as 85% of the total carbon in the *E. coli* extracellular matrix [63]. Indeed, the use of small molecule inhibitors targeting curli production can have a destructive effect on biofilm formation [64]. Outside of proteobacteria, Firmicutes such as *S. aureus* and *B. subtilis* utilize PSM’s [9] and TasA [12] amyloids as notable biofilm components. Even in eukaryotes such as fungi [22] and microalgae [23], amyloids are the conduit that give biofilms their adhesive properties.

**Figure 3:**
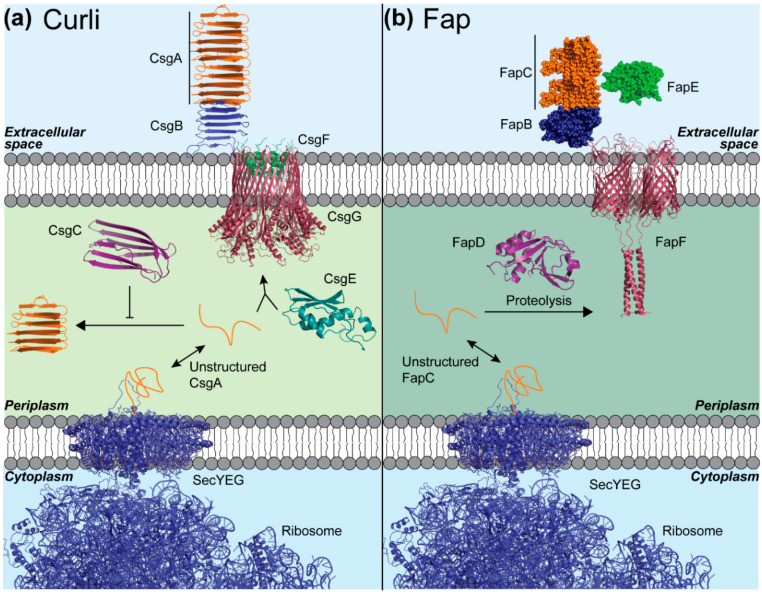
The bacterial amyloid systems called curli in *E. coli* and fap in *P. aeruginosa* are controlled through complex, multi-protein mechanisms. (**a**) The curli operon contains 7 proteins (6 shown here), each playing a necessary role in maintaining spatiotemporal control over polymerization of the major curli subunit CsgA [72]. CsgA is translated and translocated directly into the periplasm using the SecYEG secretion pore (PDB: 4V6M). CsgC (PDB: 2y2y) is a chaperone-like protein which inhibits CsgA aggregation within the periplasm. CsgE (PDB: 2NA4) is another periplasmic chaperone which fosters CsgA translocation through the nonameric curli assembly pore CsgG (PDB: 6L7A). Lastly, CsgF (PDB: 6L7A) and CsgB (REF [73]) both help to localize curli formation to the CsgG pore and the outer membrane, respectively. (**b**) In *Pseudomonas*, the major fap component FapC, is secreted into the periplasm using a SecYEG pore. FapD (modeled after the homologous C39 peptidase domain of ABC transporter PCAT1, PDB: 4RY2) is a peptidase which performs an essential proteolytic modification to one or more of the fap proteins. FapC is passed through the outer membrane using FapF, a trimeric polypeptide transporter (PDB: 5O67). Finally, FapB and FapE are essential minor components of fap amyloids, with FapB potentially playing a nucleator role similar to CsgB. Models shown of FapC, FapB, and FapE are structural predictions produced by the FALCON@home server, since there is no putative structural data in the literature.

Curli amyloids are produced in an exquisitely controlled process [5] (Figure 3a). The *E. coli* curli-specific operon (csg) contains seven proteins including the master biofilm regulator protein CsgD which regulates curli production by responding to changes in the expression of hundreds of genes and external stimuli [65]. CsgA, the major curli subunit and functional amyloid protein, is translated and translocated directly into the periplasm through the SecYEG complex on the *E. coli* inner membrane [6]. Inside the periplasm, the nascent and unfolded CsgA is stabilized in an unstructured state by a chaperone-like protein called CsgC [66]. A second chaperone, CsgE, ferries CsgA to an outer membrane pore composed of the homo-nonameric CsgG [67]. CsgA then passes through the pore to be fully secreted into the extracellular space [68]. CsgB, the curli nucleator protein, is also secreted in the same fashion [69], however, it becomes anchored to the cell surface by CsgF [70]. Finally, curli fibers form on the cell surface after extracellular CsgA amyloid formation is templated by CsgB nuclei [71]. It is through the action of all these proteins that *E. coli* can assemble amyloid fibers at the correct time and space so that cellular fitness is not compromised.

Fap is another bacterial biofilm amyloid that displays a well-controlled mechanism of formation in *P. fluorescens* and other Pseudomonads [11]. In fact, the mechanism for amyloid formation in *P. fluorescens* is quite similar to the mechanism responsible for curli production in *E. coli* [74] (Figure 3b). Fap production is controlled by a larger operon composed of 6 genes, named *fapABCDEF*, in which the dominant amyloid forming protein is FapC [75]. FapA acts as a regulator of transcription, which alters the distribution of FapB and FapC in the amyloid product [75]. FapB is a nucleator protein that assists FapC as it assembles on the outer membrane of the bacterium [11], similar to CsgA-CsgB in *E. coli* [76]. FapE is also incorporated at the end of the amyloid fibrils, potentially serving as a site for protein–protein interaction [75]. FapF forms a channel that shuttles FapB, FapC, and FapE to the outside of the cell membrane [77], like CsgG in *E. coli* [67]. The role of FapD is still a little unclear, though it has essential proteolytic activity necessary for FapC secretion [77] and may potentially be involved in cleavage of FapF [77].

## 5. Other Functional Amyloids Are also Assembled in a Controlled Manner

Yeast cells have adapted multiple ways to control the formation of amyloid fibers associated with the yeast prion Sup35 and its commonly observed phenotype [*PSI+*] [78]. Sup35 prion formation is dependent on another yeast prion called [*PIN+*], the insoluble amyloid form of Rnq1, a protein of unknown function [79]. The mechanism for the regulation of [*PSI+*] by [*PIN+*] is still unclear as it relies on an inefficient and inconsistent process called “seeding”. Seeding describes the de novo construction of one prion through the interaction with a preexisting prion [79]. Though [*PIN+*] is required for the de novo formation of [*PSI+*], [*PIN+*] is not required for the extensive propagation of [*PSI+*] [80]. In fact, once the [*PSI+*] state has been established, [*PIN+*] is no longer necessary [80]. Once amyloid formation has started, the chaperone protein Hsp104 is required for the maintenance and propagation of [*PSI+*] [81]. There exists a critical concentration of Hsp104 that is necessary for [*PSI+*] formation. Too little chaperone prevents prion formation entirely, and if the concentration of Hsp104 is too high the chaperone will dissociate from the unfolded prion intermediately and prevent proper aggregation [81]. A lack of Hsp104 has been proven to cure yeast cells of [*PSI+*] and return them to the [*psi−*] state [81]. The manner in which Hsp104 facilitates aggregation is not fully clear, though it is possible that Hsp104 cleaves the Sup35 protein into smaller fragments that are necessary for their inheritance and propagation as amyloids [44]. Sup35 aggregation is also controlled by association with Sup45, a binding partner that is essential for translation termination behavior [82]. When Sup45 is overexpressed, [*PSI+*] formation is inhibited [83].

Sup35 plays an interesting role in yeast biology, acting as a method to quickly increase genetic variation in response to swaying environmental conditions. [*PSI+*] is a yeast prion that represents the inactivated, aggregated state of Sup35, a ribosomal elongation factor [84]. When Sup35 is soluble and active, the predominant phenotype is known as [*psi−*], and the yeast ribosome correctly recognizes stop codons and terminates translation [85]. Cells can undergo a transition to the [*PSI+*] state using the controlled mechanism discussed above, which decreases nonsense suppression [86]. When yeast cells are challenged to grow under stressful growth conditions, [*PSI+*] cells are capable of creating novel, heritable phenotypes more fit to survive in the new environment [84]. The functionality of [*PSI+*] formation is controversial, as it has been argued that the resulting decrease in translational fidelity is toxic rather than beneficial [87]. While the usefulness of [*PSI+*] remains debated, there is good evidence to support the evolutionary benefits of transient decreases in translational fidelity. Additionally, there are other examples of functional amyloids in yeast, including Rim4 [88] and Cdc19 [89].

**Figure 4:**
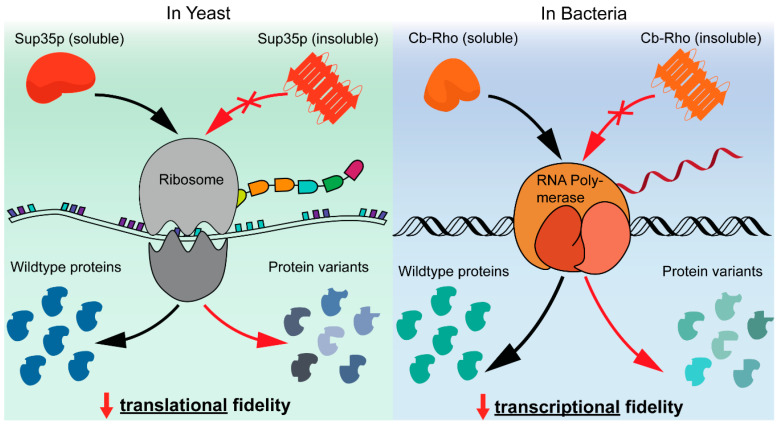
The insoluble amyloid forms of Sup35p and Cb-Rho allow the creation of protein variants better suited to survive in sudden environmental fluctuations. In yeast, the loss of an active translation termination factor Sup35p leads to a stop codon read-through, giving rise to new phenotypes. In bacteria, the same result is accomplished through the loss of the transcriptional terminator factor Rho and, thereby, a decrease in transcriptional fidelity.

In a recent publication, Yuan et al. showed that bacteria also use functional amyloids to speed up the development of new protein variants [17]. *Clostridium botulinum* has a transcription factor Rho that was found to contain a well-conserved candidate prion-like domain (cPrD) [17]. Chimeric proteins containing Cb-Rho cPrD produced phenotypes identical to [*psi−*] and [*PSI+*] in recombinant *E. coil* [17]. Interestingly, while [*PSI+*] decreases translational fidelity in yeast, Rho prions decrease transcriptional fidelity in bacteria, creating genetic variation in distinct yet similar manners (Figure 4).

Human cells have established a well-controlled mechanism to post-translationally regulate PMel17 amyloid formation in melanosomes. After synthesis, PMel17 associates with intraluminal vesicles (ILVs) of multivesicular bodies, where further processing and amylogenesis take place [90]. The tetraspanin protein CD63 ensures proper association of pre-processed PMel17 with ILVs by protecting it from degradation pathways [91]. Apolipoprotein E (ApoE) is also important for amyloid formation after CD63 has carried out its function [92]. Though its role is not fully understood, ApoE acts downstream of CD63 and likely functions to assist in the sorting of PMel17 as it associates with ILVs [92]. Once associated with ILVs, PMel17 is cleaved into two subunits, Mα and Mβ, in a specified Golgi compartment [93] by a furin-like proprotein convertase [94]. The two subunits remain connected by a disulfide bond [93] until the endosomal sheddase BACE2 catalyzes the release of the Mβ subunit from the membrane-bound Mα complex [95]. The Mβ subunit is subsequently degraded by γ-secretase activity [96]. The larger fragment, Mα, remains membrane-bound to the membranes of ILVs and acts as a nucleation site upon which amyloid formation takes place [93]. Mα is further cleaved into 3 subdomains by lysosomal proteases, fragments which form the core of PMel17 amyloids [97].

While the formation of functional amyloids is tightly regulated and predictable, pathogenic amyloid formation is stochastic and unpredictable. The inappropriate accumulation of amyloid deposits and their associated pathologies are often age-dependent processes [98,99]. Amyloid formation and the resulting protein folding diseases can be coupled to the natural decline in chaperone activity and proteosome capacity in the cell [99]. Amyloidoses most often begin with a spontaneous event during which normal proteins go above a critical concentration and transition into a pathogenic state [52]. In other cases, some amyloidoses are the result of infection. Prusiner’s protein-only theory postulated that the infective agent transferred between individuals in prion diseases were misfolded proteins [100]. Regarding sporadic Parkinson Disease, Braak’s hypothesis suggests that alpha-synuclein aggregation could be triggered by outside pathogens that introduce amyloids to distal nervous tissue [101]. Interestingly, several recent publications suggest that Braak’s pathogens could be bacterial amyloids from the microbiome [102,103,104,105]. In the case of dialysis-related amyloidosis, interventional medicine is to blame for the buildup of β2-microglobulin amyloids at needle injection sites [106]. These examples illustrate the sometimes random nature of pathogenic amyloidogenesis, which is in contrast to the controlled and predictable ways that functional amyloids form.

## 6. Conclusions

Historically, amyloids have been conceptually tied to the devastating human diseases that they can cause. However, in the last twenty years there have also been dozens of functional amyloids described that have helped usher in a new appreciation of amyloid biology. Since the amyloid conformation is a structure that is intrinsically available to all polypeptides, it is not surprising that nature has found many uses for the amyloid state. Indeed, examples of beneficial amyloids can be found all over biology, performing a wide range of tasks. Evidence of the longevity and usefulness of functional amyloids can be seen in their widespread stewardship. Where functional amyloids used to represent the exceptions in amyloid biology, they are now robustly represented and provide a template for understanding how amyloid formation can occur without causing cellular toxicity and death.

## Figures and Tables

**Table 1:** Known functional amyloids and their wide range of putative functions.

Amyloid	Gene or Protein	Amyloid Function	Species	Year	Reference
**Bacteria**					
Curli	CsgA	Biofilm formation	*E. coli*, *Salmonella* spp., and other Enterobacteriacae	2002	[6]
Microcin E492	Mcc	Toxin storage	*Klebsiella pneumoniae*	2005	[7]
Hairpins	HpaG	Virulence Factor	*Xanthanomas* spp.	2007	[8]
Phenol-soluble modulins	PSM	Biofilm formation and virulence	*Staphylococcus aureus*	2007	[9]
MTP	mtp	Pili formation	*Mycobacterium tuberculosis*	2007	[10]
FapC	FapC	Biofilm formation	*Pseudomonas fluorescens* and other Pseudomonads	2010	[11]
TasA	TasA	Biofilm formation	*Bacillus subtilis*	2010	[12]
P1	P1	Adhesin	*Streptococcus mutans*	2012	[13]
Listeriolysin	LLO	Phagolysosome	*Listeria monocytogenes*	2012	[14]
RepA	RepA	Plasmid replication regulator	*Pseudomonas aeruginosa*	2016	[15]
Chaplins	ChpA-H	Decreasing surface tension	*Streptomyces coelicolor*	2017	[16]
Rho	Rho	Transcriptional regulator	*Clostridium botulinum*	2017	[17]
**Protists**					
MSP2	msp2	Erythrocyte invasion	*Plasmodium falciparum*	2009	[18]
**Fungi**					
Het-s	het-s	Heterokaryon formation	*Podospora anserina*	1997	[19]
Ure2 ^†^	Ure2	Nitrogen catabolism regulator	*Saccharomyces cerevisiae*	1997	[20]
Sup35 ^†^	Sup35	Translation regulator	*Saccharomyces cerevisiae*	1997	[20]
Hydrophobins	SC3, etc	Breaking water surface tension	*Schizophyllum commune*, *basidiomycetes*, etc.	2000	[21]
Adhesions	Als Proteins	Biofilm formation	*Candida albicans* and other fungi	2004	[22]
**Plants**					
Adhesive substance	unknown	EPS ^‡^ component	*Coccomyxa* spp., *Glaphyrella trebouxiodes*, and other microalgae	2008	[23]
Rubber elongation factor	HevB1	latex biosynthesis	*Hevea brasiliensis*	2012	[24]
Defensins	RsAFP-19	Antifungal defense	*Raphanus sativus*	2013	[25]
AMP2	Cn-AMP2	Antimicrobial defense	*Cocos nucifera*	2016	[26]
**Animals**					
Chorions	Chorions	Egg protection	*Bombyx mori*, *Papilio xuthus*, etc.	2000	[27]
Spidroin	Spidroin	Silk production	*Nephila edulis*, *Araneus diadematus*, etc.	2002	[28]
CPEB	CPEB	Translation regulator	*Aplysia californica*	2003	[29]
Pmel17	Pmel17	Melanin synthesis	*Homo sapiens*	2005	[30]
Cement	cp-100k	surface adhesion	*Megabalanus rosa* and other barnacles	2006	[31]
Peptide hormones	GLP-2, VIP, etc.	Storage	*Homo sapiens*	2009	[32]
Anionic dermaseptin	aDrs	host defense	*Pachymedusa dacnicolor*	2012	[33]
Orb2	Orb2	Memory persistence	*Drosophila melanogaster*	2012	[34]
epididymal cystatin	cst8, etc.	Sperm maturation	*Mus musculus*	2012	[35]
RIP kinases	RIP1/3	Necrosis regulator	*Homo sapiens*	2012	[36]
Uperin 3.5	uperin 3.5	Antimicrobial defense	*Uperoleia mjobergii*	2016	[37]
**Archaea**					
Biofilm amyloid protein	HVO_143	Biofilm formation	*Haloferax volcanii*	2014	[38]

^†^ Only the two first yeast prions that were identified are mentioned here. This is not an inclusive list; there are many more examples of prions performing important functions in yeast. ^‡^ Extracellular polymeric substance.
